# Esophageal Cancer in Elderly Patients, Current Treatment Options and Outcomes; A Systematic Review and Pooled Analysis

**DOI:** 10.3390/cancers13092104

**Published:** 2021-04-27

**Authors:** Styliani Mantziari, Hugo Teixeira Farinha, Vianney Bouygues, Jean-Charles Vignal, Yannick Deswysen, Nicolas Demartines, Markus Schäfer, Guillaume Piessen

**Affiliations:** 1 Department of Visceral Surgery, Faculty of Biology and Medicine UNIL, Rue du Bugnon 46, Lausanne University Hospital (CHUV), 1011 Lausanne, Switzerland; hugo.teixeira-farinha@chuv.ch (H.T.F.); demartines@chuv.ch (N.D.); markus.schafer@chuv.ch (M.S.); 2 Department of Digestive Surgery, CH Saint Brieuc, 10 Rue Marcel Proust, 22000 Saint-Brieuc, France; vianney.bouygues@ch-stbrieuc.fr; 3 Department of Visceral Surgery, Clinique Chirurgicale Du Libournais, 119 Rue de la Marne, 33500 Libourne, France; docteur.jcvignal@gmail.com; 4 Clinique Universitaire St Luc, Université Catholique de Louvain, Bruxelles, Belgium Avenue Hippocrate 10, 1200 Bruxelles, Belgium; yannick.deswysen@chu-bruxelles.be; 5 Department of Digestive and Oncological Surgery, Claude Huriez University Hospital, University of Lille, F-59000 Lille, France; guillaume.piessen@chru-lille.fr

**Keywords:** esophageal cancer, elderly, surgery, esophagectomy, definitive chemoradiation

## Abstract

**Simple Summary:**

Any given treatment may provide improve survival for elderly patients with oesophageal cancer compared to best supportive care. Although surgery may be related to a higher rate of complications in these patients, it also offers the best chance for survival, especially when combined with perioperative chemo-or chemoradiation. Definitive chemoradiation remains also a valid and widely used curative approach in this population. Quality of life after oesophageal cancer treatment does not seem to be particularly compromised in elderly patients, although the risk of loss of autonomy after the disease is higher. Based on the available data, excluding a priori elderly patients from curative treatment based on age alone cannot be supported. A thorough general health status and geriatric assessment is necessary to offer the optimal treatment, tailored to the individual patient.

**Abstract:**

Esophageal cancer, despite its tendency to increase among younger patients, remains a disease of the elderly, with the peak incidence between 70–79 years. In spite of that, elderly patients are still excluded from major clinical trials and they are frequently offered suboptimal treatment even for curable stages of the disease. In this review, a clear survival benefit is demonstrated for elderly patients treated with neoadjuvant treatment, surgery, and even definitive chemoradiation compared to palliative or no treatment. Surgery in elderly patients is often associated with higher morbidity and mortality compared to younger patients and may put older frail patients at increased risk of autonomy loss. Definitive chemoradiation is the predominant modality offered to elderly patients, with very promising results especially for squamous cell cancer, although higher rates of acute toxicity might be encountered. Based on the all the above, and although the best available evidence comes from retrospective studies, it is not justified to refrain from curative treatment for elderly patients based on their age alone. Thorough assessment and an adapted treatment plan as well as inclusion of elderly patients in ongoing clinical trials will allow better understanding and management of esophageal cancer in this heterogeneous and often frail population.

## 1. Introduction

Esophageal cancer is the 9th most common cancer worldwide [1,2], and one of the deadliest malignancies, with a European mean 5-year survival of 12%, according to the EUROCARE-5 study [3]. Although long-term survival has been markedly improved in recent years, especially for non-metastatic stages, overall prognosis remains poor. Its peak incidence has shifted from 65–70 to 70–79 years [4,5], with 33% of patients being over 75 and 8% over 85 years old upon diagnosis [4]. Thus, with the global increase of life expectancy and the improving diagnostic and therapeutic means, health care providers will increasingly encounter this disease among the aging population.

Surgery in elderly patients (EP) with esophageal cancer was assessed by two meta-analyses in patients >70 years [6] and >80 years [7], reporting increased postoperative morbidity and shorter overall survival compared to younger patients (YP). Both meta-analyses included the same primary studies, some published up to 30 years ago. Moreover, evidence is scarce for definitive chemoradiation or multimodal treatment in EP, although they are currently integral part of esophageal cancer management [1,5]. Of prime importance is health-related quality of life and loss of autonomy in EP treated for esophageal cancer, as these are outcomes just as (if not more) important than survival per se in the aging population [8]. Thus, the clinician treating EP with esophageal cancer needs to be acquainted with all of the above facets to offer optimal management to this fragile group of patients.

The aim of this systematic review was to summarize published data on all types of curative treatment, including surgery, multimodal treatment, definitive chemoradiation and local ablation, as well as the impact of treatment on the quality of life (QoL) of EP with esophageal cancer.

## 2. Methods

### 2.1. Search Strategy and Selection Criteria

Five authors (SM, HT, VB, JCV, YD) independently undertook electronic literature searches with Medline via PubMed and Embase. The detailed research strategy for Pubmed is shown in online Appendix A. The PRISMA reporting guidelines for systematic reviews were followed for this study [9].

Eligible for inclusion were studies that defined EP as at least ≥70 years old upon diagnosis and assessed any kind of curative treatment of esophageal cancer including surgery (S), multimodal treatment (neoadjuvant treatment and surgery (NATS)), definitive chemoradiation (DCRT), local ablation. Studies on palliative treatment as well as those reporting on ‘gastroesophageal cancer’ without distinct analysis of esophageal cancer were excluded.

Randomized controlled trials and cohort studies published between January 2000 and March 2020 were eligible for this review, if they were comparative between elderly (EP) and younger patients (YP) for a certain type of treatment or between EP for different treatment types. Only articles in English were included in the search. Non-comparative studies, editorials, case reports, reviews, conference abstracts and opinion articles were excluded. The reference lists of selected articles and relevant review articles were also hand searched for additional papers.

### 2.2. Data Management, Risk of Bias Assessment

Two authors (SM and HT) independently screened full-text articles and extracted data in an Excel datasheet, based on the Cochrane sample of good practice [10]. Study quality and risk of bias was assessed by means of the Newcastle-Ottawa Scale [11] for non-randomized trials, and studies with a minimum of 6 points were included in the review. Pooled analysis was used for quantitative summary of results for each outcome.

## 3. Results

**Figure 1:**
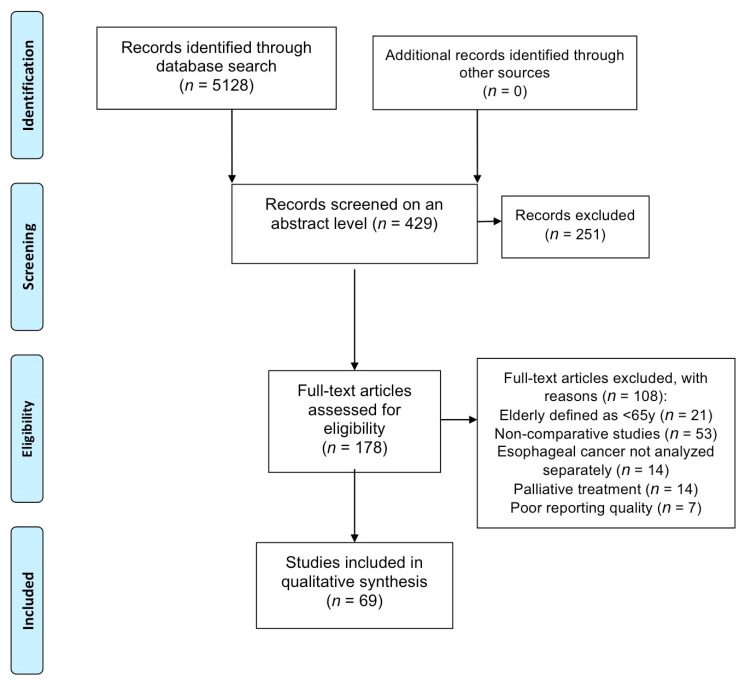
The flowchart of study selection according to the PRISMA guidelines [9].

As shown in the study flowchart (Figure 1), 69 studies were retained for this systematic review, including 82,531 elderly patients (EP). All articles were cohort studies. Thirty-nine studies defined EP as >70 years, 15 as >75 years and 15 studies as ≥80 years old. Across these studies, the median percentage of patients >70 years was 28% (range 3–66), whereas 17% of patients (range 8–45) were >75 years and 10% (range 2–50) > 80 years old. None of the studies revealed sex-related differences in incidence or treatment of esophageal cancer.

### 3.1. Access to Curative Treatment for Elderly Patients (EP)

Several authors investigated the accessibility of EP to different treatment options [12,13,14,15,16,17,18]. Among EP with curable disease, only 50–69% seemed to receive treatment with curative intent [12,13,15,16]. Münch et al. even report that for similar tumor stages with YP, 25% of EP received palliative and not curative treatment [19]. The most frequently used modality was definitive chemoradiation (DCRT) in 37–64% of EP [12,13,14], while only 7–36% of them were offered surgery [12,13,15,16,17,18].

However, in all of the above studies any given treatment offered a substantial survival benefit for EP [12,13,15,16,17,18]. Median overall survival (OS) after curative treatment was 18–19 months, 6–9 months longer than palliative strategies [13,16]. The best results are reported after neoadjuvant treatment and surgery (NATS), followed by upfront surgery and DCRT [12]; indeed, surgery compared to non-surgical treatment increased median OS from 5–10 to 18–22 months [17,18], and 5-year OS from 3% to 23% [15]. Even palliative radiation or chemotherapy offered a 3-to 8-month survival benefit compared to best supportive care (7–10 vs. 2–4 months) [12,16]. Of note, the decision to refrain from curative treatment comes from the physician in 46% and from the patient in 46% [13].

In summary, although EP have limited access to curative treatment of esophageal cancer, they can obtain a substantial survival benefit by any treatment modality compared to best supportive care.

### 3.2. Comparison of Different Treatment Strategies Offered to EP

#### 3.2.1. Surgery Alone Versus Neoadjuvant Treatment and Surgery (NATS) in EP

Three studies assessed postoperative outcomes and survival in EP treated by surgery only or NATS [20,21,22].There were no differences in anastomotic leakage rate (15–18% for surgery vs. 10–20% for NATS) [21,22], pulmonary complications (15–25% for surgery vs. 20–35% for NATS) [21,22] or postoperative mortality (12–14% vs. 10–12% respectively) [20,21]. Rice et al. reported higher postoperative cardiovascular complications, mainly atrial fibrillation, in EP treated with NATS (34% vs. 18%, *p* = 0.008) [22]. This finding was not confirmed by Camerlo et al., who found similar rates (9–10%) of cardiovascular complications for EP with surgery and NATS [21]. One study reported significantly better median overall survival for NATS versus surgery only in EP [20], whereas the difference was not significant in the study by Camerlo et al. (21 vs. 22 months, *p* = 0.807) [21]. In summary, there is no sufficient evidence to support increased postoperative morbidity or mortality for EP after NATS.

#### 3.2.2. Surgery Versus Definitive Chemoradiotherapy (DCRT) in EP

Four studies assessed long-term survival in EP treated with surgery versus DCRT [15,23,24,25]. Jing et al. report superior overall survival for EP treated with surgery (36 vs. 15 months, *p* < 0.001), whereas progression-free survival, was also significantly better for surgery compared to DCRT (27 vs. 12 months, *p* < 0.0001) [23]. Faiz et al. also found longer median survival after surgery (19 vs. 15 months, *p* < 0.001) [15]. This finding is confirmed by Kosugi et al. (22 vs. 13 months), although in this study EP treated with DCRT seemed to have more advanced disease (cT4 tumors) than those treated with surgery [24]. Takagawa et al. found a 7-month longer median survival after surgery compared to DCRT (22 vs. 15 months) [25].

In summary, EP with curable esophageal cancer treated with surgery have superior survival than those treated with DCRT. Caution is needed as DCRT patients often have more advanced tumor stage, and the use of salvage surgery remains unknown in this population.

### 3.3. Surgery for Esophageal Cancer in EP

Access to surgery was limited for EP compared to YP in all 8 relevant studies, with a median of 38% (range 12–76%) EP vs. 58% (range 14–86%) YP being offered curative surgery for similar tumor stages [18,25,26,27,28,29,30,31]. This difference was statistically significant in six studies [18,26,27,28,29,30]. Furthermore, Faiz et al. report a significant reduction of the rate of surgery among EP, as age increases; from 18.3% for patients 75–80 y, it is reduced to 6.4% among 80–85 y and 2.3% for patients >85 y (*p* < 0.001) [15]. Thirty-five studies assessed short-term (Table 1) and long-term (Table 2) outcomes of surgery in EP compared to YP.

#### 3.3.1. Anastomotic Leakage

Among the 19 studies assessing anastomotic leakage for EP versus YP, none revealed a difference in the incidence of this complication (Table 1) [14,30,31,32,33,34,35,36,37,38,39,40,41,42,43,44,45,46,47]. Anastomotic leakage rates presented considerable variability, reaching 25–37.5% for series with cervical anastomoses [31,35,43]. Schweigert et al. reported a significantly increased postoperative mortality (OR = 8.24, *p* = 0.025) for EP compared to YP who present an anastomotic leakage [37].

#### 3.3.2. Pulmonary Complications

Twenty-three studies assessed postoperative pulmonary complications, with a median incidence of 20% (range 4.2–62.9%) for EP versus 16% (range 5.1–62.2%) for YP (Table 1) [30,31,32,33,34,36,37,38,39,40,41,42,43,44,45,46,47,48,49,50,51,52,53]. The most frequently reported pulmonary complication was pneumonia, which EP presented in significantly higher rates than their younger counterparts [31,33,34,37,38,40,48,50,52]. Tapias et al. reported a higher rate of pneumonia (31.3 vs. 10.8, *p* = 0.039), but also greater risk of respiratory failure for EP compared to YP (18.8% vs. 3%, *p* = 0.002); in this study, all types of medical complications were higher for EP [38]. Liu et al. reported jointly ‘cardiopulmonary morbidity’, which was significantly higher for EP (30.8 versus 6.8%), although this did not affect postoperative mortality [50].

#### 3.3.3. Cardiovascular Complications

Eighteen studies assessed cardiovascular complications after esophagectomy for EP (Table 1), with the most frequently reported event being cardiac arrythmia/atrial fibrillation [14,18,31,32,33,34,36,38,40,41,42,43,45,46,47,48,52,53]. The median incidence of cardiovascular complications was 15.6% (range 0–38.3) for EP and 7.0% (range 0–21) for YP, the difference was significant in 8 studies with EP having approximately twice the rate of YP [33,34,38,40,41,46,48,52]. Pultrum et al. found a higher rate of postoperative fibrillation in EP (36%vs 16%, *p* < 0.001) even though baseline cardiopulmonary morbidity was similar with YP [41]. Sabel et al. report a significantly higher rate of postoperative myocardial infarction (8 versus 0%, *p* < 0.001), while atrial fibrillation was similar (23 vs. 13%, *p* < 0.05) [18].

#### 3.3.4. Postoperative Mortality

Among the 32 studies assessing postoperative mortality, median rate was 7.9% (range 0–24%) for EP compared to 3.4% (range 0–9%) for YP. In 16 of these studies, mortality was significantly higher for EP [25,29,32,34,37,39,40,43,44,47,48,54,55,56,57,58] (Table 1). Schlottmann et al. reported an almost two-fold risk of postoperative death for EP, whereas predicted mortality rate showed a steady increase with advancing age; from 1.5% for a 40-year-old patient, 3.6% in 60 years and 7% for patients >80 years [48]. Elsayed et al. found age >70 years to be independently associated with postoperative death, along with impaired baseline cardiac and pulmonary function [39]. On the contrary, Paulus et al. found no difference in postoperative morbi-mortality after matching EP and YP for baseline comorbidity [36], whereas in the study of Markar et al., even though EP were >80 years with higher baseline cardiopulmonary comorbidity and more postoperative complications, 90-day mortality remained very low (0%vs 0.6%, *p* = 0.99) [52].

**Table 1:** Postoperative outcomes after curative surgery for esophageal cancer in EP vs. YP.

Author, Year [Reference]	EP Definition(Years)	No of EP Patients (% to All)	Anastomotic Leakage (%) EP/YP	Pulmonary Complications (%) EP/YP	Cardiovascular Complications (%) EP/YP	Postoperative Mortality (%) EP/YP	
Aoyama, 2020 [43]	75	24 (24.5)	37.5/33.7	33.3/27.5	12.6/6.1	4.2/0 *	30-day
Song, 2020 [58]	79	35 (3.5)	-	-	-	5.8/5.7 *	90-day
Chen, 2019 [44]	70	142 (25.8)	17.6/10	4.2/7.6	-	2.1/2.9	In-hospital
Chen, 2019 [53]	80	34 (21.7)	-	29/27	3/2	5/2 *	90-day
Kanda, 2019 [45]	75	59 (15.8)	4/3	20/16	12.5/7.5	-	-
Baranov, 2019 [46]	75	89 (24.9)	19.1/17.1	62.9/62.2	24.7/14 *	9/5	90-day
Klevebro, 2018 [47]	75	62 (16.9)	8.3/10.7	20/19.7	6.7/5.7	18.6/7.6 *	90-day
Schlottmann, 2018 [48]	70	1544 (29.5)	-	25/20 *	9/4 *	8.9/4.3 *	In-hospital
Paulus, 2017 [36]	80	33 (50)	18/12	18/6	24/21	18/9	90-day
Miyata, 2015 [49]	70	213 (30)	-	17/14	-	0/0.4	In-hospital
Liu, 2015 [50]	70	39 (21)	-	30.8/6.8 * (♯)		7.7/3.4	-
Stahl, 2014 [56]	80	289 (-)	-	-	-	8/2.1 *	-
Morita, 2013 [51]	80	104 (10)	-	13/17.7	-	13/4.8	In-hospital
Mirza, 2013 [59]	70	46 (22)	-	-	-	11/9	30-day
Schweigert, 2013 [37]	75	45 (15)	13/13	53/30 *	-	24/9 *	In-hospital
Tapias, 2013 [38]	80	16 (3.4)	0/4.8	31.3/10.8 *	37.5/14.4 *	-	-
Markar, 2013 [52]	80	32 (6.4)	-	18.8/8.3 *	31.3/16.7 *	0/0.6	90-day
Cijs, 2010 [29]	70	564 (29)	-	-	-	11/5.4 *	In-hospital
Elsayed, 2010 [39]	70	108 (33.1)	5/9	32.5/37	-	12/5 *	In-hospital
Yang, 2010 [40]	70	136 (50)	6.6/4.4	17.6/8.1 *	24.2/14 *	5.9/0.7 *	-
Pultrum, 2010 [41]	70	64 (27.4)	13/18	56/42	36/16 *	-	-
Davies, 2010 [14]	70	59 (21)	5/6	-	3.4/1.4	7/2.4	-
Alibakshi, 2009 [42]	70	165 (34.4)	7.2/7.9	10.3/9.8	6/4.4	3/2.8	In-hospital
Takagawa, 2008 [25]	75	19 (8.6)	-	-	-	15.8/3 *	In-hospital
Morita, 2008 [57]	80	16 (2.4)	-	-	-	6/2 *	In-hospital
Ruol, 2007 [30]	70	159 (21.5)	7.5/10.2	17/15.3	-	1.9/2.7	In-hospital
Finlayson, 2007 [54]	80	3150 (11.3)	-	-	-	19.9/8.8 *	In-hospital
Moskovitz, 2006 [32]	80	31 (3.6)	6.5/8.8	9.7/4.7	0/0.8	19.4/7.3 *	In-hospital
Ma, 2006 [33]	70	60 (3.3)	3.3/2	43.4/28.1 *	38.3/19.8 *	3.3/1.1	In-hospital
Di Martino, 2005 [55]	70	51 (46.8)	-	-	-	12.1/4.1 *	In-hospital
Rahamim, 2003 [34]	70	199 (33.4)	4.5/6.1	10.1/5.1 *	15.6/7 *	12.1/5 *	30-day
Sabel, 2002 [18]	70	147 (36)	-	-	23/13 (♯♯)8/0 *(†)	4/2	In-hospital
Fang, 2001 [35]	70	79 (17.9)	26.6/35.1	-	-	7.6/3.3	In-hospital
Kinugasa, 2001 [31]	70	55 (26.9)	18.2/12.8	45.5/19.5 *	20/8.1	10.9/5.4	60-day
Johansson, 2000 [60]	70	48 (40)	-	-	-	0/2.8	In-hospital

EP = Elderly patients, YP = younger patients. Significant *p*-values < 0.05 are indicated with an asterisk *. (♯) Reported jointly as ‘cardiopulmonary morbidity’, (♯♯) rates of atrial fibrillation, (†) rates of infarctus.

#### 3.3.5. Surgical Approach, Quality of Surgery

Three studies report results of different surgical approaches in EP. Li et al. compared minimally invasive to open esophagectomy in EP, and found a significant reduction of both overall (37.9% vs. 60.3%, *p* = 0.016) and pulmonary complications (20.7% vs. 39.7%, *p* = 0.0026) with the minimally invasive approach [61]. Liu et al. [62] compared the Ivor Lewis procedure to a left-sided transthoracic approach (Sweet procedure), and found significantly less pulmonary complications (22.8 vs. 8.8%, *p* = 0.04) and a lower infra-clinic leakage rate (14 vs. 1.8%, *p* = 0.032) with the left-sided approach. However, lymph node yield was significantly better for the Ivor Lewis procedure and so was overall (5-year 54.6 vs. 32.6%, *p* = 0.036) and disease-free survival (5-year 52.7 vs. 20.2%, *p* = 0.021). Chen et al. compared the McKeown with the Sweet procedure in EP, the latter being associated with less anastomotic leaks (11.3% vs. 23.9 vs, *p* = 0.038) [44].

Several other studies compared the surgical approach offered to EP compared to YP with esophageal cancer. Five authors reported a significantly lower rate of transthoracic resections for EP [25,33,37,63,64], probably reflecting the widespread idea of avoiding thoracotomy to reduce pulmonary and overall complications. Schweigert et al. still found significantly more pulmonary complications (53% vs. 30%, *p* = 0.003) and mortality (24% vs. 9%, *p* = 0.008) for EP compared to YP, even though a transhiatal approach was more frequently used in the EP group (16% vs. 4%, *p* = 0.005) [37]. Takagawa et al. also report higher postoperative mortality (15.8 vs. 3% *p* = 0.032) for EP, although a transthoracic approach was used in only 68% of them (versus 91% in YP, *p* = 0.009) [25]. In the study of Ma et al., although only 68% of EP versus 88% of YP had a thoracotomy (*p* < 0.0001), pulmonary complications remained higher for the EP group (43.4 vs. 28.1% respectively, *p* = 0.01) [33]. Only one study identified transthoracic approach (along with respiratory comorbidity and neoadjuvant treatment) as an independent risk factor of postoperative mortality in EP [29]. Also of note, among the studies where a transhiatal approach was preferred for EP, patients had consistently a shorter overall survival compared to YP [25,63,64].

In the four studies reporting the quality of surgery performed to EP compared to YP, there were no significant differences in terms of R0 resection [25,46,53,55]. Zehetner et al., however, reported less radical lymphadenectomy for EP (median resected lymph nodes = 24 vs. 37, *p* < 0.0001) [64].

#### 3.3.6. Long-Term Survival after Surgery

Thirty studies assessed long-term survival among EP compared to YP treated by surgery for esophageal cancer (Table 2). Fourteen studies found a significantly shorter overall survival for EP [25,29,32,34,36,38,40,43,49,54,57,58,64,65], among whom 8 concern EP ≥ 80 years. Pooled 5-year overall survival was estimated to 29.3% (9–42.9%) for EP versus 35.1% (21–64.8%) for YP, and median survival was 20 months (range 10.8–53.2 months) versus 29.6 months (range 13.7–77.6 months), respectively.

Eleven studies reported disease-free or disease-specific survival (Table 2). Only four authors found a disease-free survival advantage for YP [29,38,43,49], suggesting that no robust suggestions for earlier cancer recurrence in EP can be made.

In summary, no evidence exists for higher rates of postoperative anastomotic leakage for EP, although pulmonary and cardiovascular complications as well as postoperative death occur more frequently in these patients. Minimally invasive esophagectomy may reduce pulmonary complications for EP, whereas avoiding thoracotomy with a transhiatal approach does not offer a benefit on postoperative morbidity. Overall long-term survival is shorter in EP, but data on disease-specific survival are far less conclusive.

**Table 2:** Long-term survival after curative surgery for esophageal cancer in EP vs. YP.

Author, Year [Reference]	EP Definition (Years)	Disease-Related Survival EP/YP		Overall Survival EP/YP	
Aoyama, 2020 [43]	75	15.1/20.7 *	Median DFS, mo	16.4/29.8 *	Median (mo)
Song, 2020 [58]	79	-	-	18/62 *	Median (mo)
Chen, 2019 [44]	80			38.5/40.4	Median (mo)
Bakhos, 2019 [65]	80	-	-	23/29.3 *	Median (mo)
Kanda, 2019 [45]	75	12/33	5-year DFS (%)	12/35	5-year (%)
Baranov, 2019 [46]	75	-	-	57.3/54.5	2-years (%)
Paulus, 2017 [36]	80	-	-	40/62 *	5-year (%)
Miyata, 2015 [49]	70	42/58*	5-year DFS (%)	29.3/52.4 *	5-year (%)
Liu, 2015 [50]	70	-		15.8/13.7	Median (mo)
Morita, 2013 [51]	80	38/40	5-year DFS (%)	10/35	5-year (%)
Mirza, 2013 [59]	70	-		10.8/15.6	Median (mo)
Tapias, 2013 [38]	80	49.2/72.4 *	5-year DSS (%)	49.2/64.8 *	5-year (%)
Markar, 2013 [52]	80	-		53.2/77.6	Median (mo)
Cijs, 2010 [29]	70	27/34 *	5-year DSS (%)	22/29 *	5-year (%)
Zehetner, 2010 [64]	80	48.9/57.3	Median, DSS (mo)	19.7/42.5 *	Median (mo)
Elsayed, 2010 [39]	70	-		20/27.6	Median (mo)
Yang, 2010 [40]	70	24/35.5	5-year DFS (%)	30/41.8 *	5-year (%)
Pultrum, 2010 [41]	70	-		33/33	5-year (%)
Davies, 2010 [14]	70	-		20/28	Median (mo)
Takagawa, 2008 [25]	75	40/46	Median, DFS (mo)	22/38 *	Median (mo)
Morita, 2008 [57]	80	22/46	Median, DFS (mo)	9/39 *	5-year (%)
Ruol, 2007 [30]	70	-		35.4/33.6	5-year (%)
Finlayson, 2007 [54]	80	-		17.6/31.4 *	5-year (%)
Moskovitz, 2006 [32]	80	-		16.8/48 *	Median (mo)
Di Martino, 2005 [55]	70	-		17.8/35.1	5-year (%)
Rahamim, 2003 [34]	70	-		13/21 *	5-year (%)
Sabel, 2002 [18]	70	-		24/27	Median (mo)
Fang, 2001 [35]	70	55.4/59.1	5-year (%), DSS	40.9/48.1	5-year (%)
Kinugasa, 2001 [31]	70	-		32.9/35.3	5-year (%)
Johansson, 2000 [60]	70	-		24/24	Median (mo)

EP = Elderly patients, YP = Younger patients, DFS = Disease-Free Survival, DSS = Disease-specific survival. Significant *p*-values < 0.05 are indicated with an asterisk *.

### 3.4. Neoadjuvant Treatment Followed by Surgery (NATS)

As observed above for surgery, NATS was less frequently offered to EP, with a pooled median rate of 20% (range 1–84%) compared to 46% of YP (range 4–91%). The difference was significant in all studies [27,29,30,31,32,33,34,37,38,49,52,59,64,66,67] but one [39]. Vallböhmer et al. reported that even for cT3-T4 disease with a clear indication for NATS, only 28% of EP received neoadjuvant treatment compared to 81% of YP (*p* < 0.0001) [67].

#### 3.4.1. Postoperative Complications and Mortality Following NATS

Table 3 summarizes the main postoperative outcomes after NATS for EP in comparison to YP. Braiteh et al. [68] reported a significantly higher rate of anastomotic leakage for EP compared to YP (11.3 vs. 6.3%, *p* = 0.047), although none of the other studies confirm this finding. This was also the only study to report higher respiratory complications after NATS for EP (32 vs. 27%, *p* = 0.045), whereas four studies found significantly higher cardiovascular complications for EP compared to YP after NATS [22,63,68,69]. Two studies report no difference in postoperative outcomes between EP and YP [21,70], but none of the retrieved studies reported higher mortality rates for EP treated with NATS.

#### 3.4.2. Histologic Response to Neoadjuvant Treatment

Vöncken et al. [27] reported a significantly higher rate of complete response to treatment in EP compared to YP (50% vs. 25%, *p* = 0.02), whereas three other studies found no significant difference [21,67,69].

#### 3.4.3. Long-Term Survival after NATS

Among the seven studies reporting long-term overall survival after NATS, only two [67,68] found a significantly reduced survival for EP; disease-free survival was reported in two studies [21,27], with no significant difference between EP and YP (Table 3). Furlong et al. [71] assessed overall survival after neoadjuvant chemoradiation (5FU-Cisplatin and 40 Gy regimen) in EP, with or without surgery. In this study, 45% of patients (all >70 y) presented a clinical complete response (57% for squamous cell and 36% for adenocarcinoma subtype). For EP with a complete response to chemoradiation surgery did not add a survival benefit, however EP with incomplete histologic response had significantly superior survival when surgery was performed after chemoradiation (median 36.2 vs. 7.9 months, *p* = 0.0006).

In summary, significantly fewer EP are offered multimodal treatment (NATS) compared to YP, even though histologic response is at least as good as YP. Cardiovascular and to a lesser degree pulmonary complications are more frequent in EP after NATS. Surgery after neoadjuvant chemoradiation may offer a significant survival benefit to EP with incomplete clinical response to treatment.

**Table 3:** Neoadjuvant treatment (NAT) and surgery for oesophageal cancer, in elderly patients (EP) versus younger patients (YP).

Author, Year [Reference]	EP Definition(years)	No of Patients	NAT Details	pCR	Anastomotic LeakAge (%)	Pulmonary Complications (%)	Cardiovascular Complications (%)	Postop Mortality (%)	Disease-Free Survival	Overal Survival
		EP (%)		EP/YP	EP/YP	EP/YP	EP/YP	EP/YP	EP/YP	EP/YP
Vöncken, 2018 [27]	70	76 (30)	5FU/Cisplatin +50 Gy or Carbo/Taxol + 41 Gy	50/25 *	-	-	-	-	22/29 median (mo)	26/38 Median (mo)
Blom, 2013 [63]	75 y	17 (8)	Carbo/Taxol	-	12/11	18/25	41/14 *	0/2	-	31/59 3-year (%)
Camerlo, 2012 [21]	70 y	52 (44)	5FU/Cisplatin + 45 Gy	10/21	10/4	35/27	10/7	10/7	22/29 Median (mo)	23/44 Median (mo)
Fogh, 2011 [70]	70 y	57 (22)	5FU/Cisplatin or 5FU/Taxol + 45–61 Gy	-	14/12	18/17	25/15	7/5	-	-
Braiteh, 2009 [68]	70 y	341 (52)	-	-	11/6 *	32/27 *	21/12 *	-	-	36/42 * Median (mo)
Vallböhmer, 2008 [67]	70 y	52 (23)	5FU/Cisplatin + 36 Gy	33/19	-	-	-	-	-	27/61 * 5-year (%)
Ruol, 2007 [69]	70 y	31 (4)	5FU/Platin+ 45–50 Gy	17/22	7/9	23/15	23/5 *	7/2		24/23 Median (mo)
Rice, 2005 [22]	70 y	35 (11)	5FU-cisplatin + 45–50 Gy	-	20/9	20/24	34/15 *	3/4	-	34/42 Median (mo)

NAT = Neoadjuvant treatment, EP = Elderly patients, YP = Younger patients, pCR = pathologic complete response. Significant *p*-values < 0.05 are indicated with an asterisk *.

### 3.5. Definitive Chemoradiation (DCRT)

As seen earlier, DCRT is the preferred curative treatment modality offered to EP. Vöncken et al. report a significantly higher rate of EP receiving DCRT compared to YP (46% vs. 28%, *p* = 0.01), whereas in the same study EP received less often both surgery and NATS [27].

#### 3.5.1. Treatment Toxicity

As illustrated in Table 4, severe (≥grade III) hematologic toxicity (leucopenia, thrombocytopenia, anemia) was reported in similar rates for EP (4–22%) and YP (12–29%) by three authors [14,19,72]. Only Takeuchi et al. found significantly higher toxicity for EP (70 vs. 49.7%, *p* = 0.042), with rates being particularly high for both groups in this study after a 5FU-Cisplatin/60 Gy regimen [73]. Severe acute pulmonary toxicity (≥grade III) was reported by only one study and was found significantly higher for EP (11 vs. 0%, *p* = 0.003); of note, in that study all patients were treated more than a decade ago, some with traditional 3D therapy [72]. Esophagitis ≥ grade III was assessed in three studies, with no significance difference found between EP (3–22.5%) and YP (7–23.5%) [14,72,73]. Tanisada et al. also report similar rates of overall acute and late treatment toxicity between EP and YP [74].

#### 3.5.2. Clinical Response to Treatment

Four studies provide rates of complete clinical response to treatment, for EP (ranging from 48–78%) and YP (31–67%) [25,27,72,73]. Among them, only Xu et al. report a significant difference, 78% vs. 56%, *p* = 0.004), in favor of EP, in a series where SCC was predominant [72]. Although rates of SCC histology varied from 29–100% (Table 4), no significant differences in histologic type (SCC/adenocarcinoma) were observed between EP and YP in all included studies.

#### 3.5.3. Comparison of DCRT Modalities in EP

Zhao et al. [75] compared DCRT to radiation only, and report higher rates of severe esophagitis (5.8% vs. 1.4%) and hematologic toxicity (9.8% vs. 0%, *p* < 0.05) in the DCRT group. However, DCRT was also associated with higher rates of clinically complete response to treatment (34.6% vs. 18.6%, *p* = 0.044) as well as median overall (24.6 vs. 19.4 months, *p* = 0.018) and progression-free survival (15.3 vs. 10.6 months, *p* = 0.008)

Two other studies [61,76] compared concurrent DCRT to sequential DCRT to radiation only. Li et al. found significantly better median overall survival for concurrent DCRT (22.3 months) compared to sequential DCRT (18 months) and radiation (12.4 months) (*p* = 0.044), while acute toxicity and notably esophagitis did not present significant differences [61]. Lu X et al. report a 5-year overall survival of 11.1% for concurrent DCRT, compared to 10.6% for reduced DCRT and 0% for radiation only (*p* < 0.001) [76]. In this study, reduced DCRT was associated with a significantly lower rate of severe acute toxicity (14.9%) compared to radiation (18.1%) and concurrent DCRT (30.3%) [76].

#### 3.5.4. Long-Term Survival

Overall survival after DCRT for EP versus YP was assessed by all eight studies [14,19,25,27,72,73,74,77] (Table 4). Vöncken et al. report a significantly better median OS for EP versus YP (23.6 months vs. 13.1 months, *p* = 0.01) who received a Carboplatin-paclitaxel/50 Gy regimen [27], whereas Takagawa et al. found a trend towards better overall survival in EP (median 15 vs. 10 months, *p* = 0.073) after a 5FU-60 Gy regimen [25]. Four studies found no significant differences [14,19,72,74]. Tanisada et al. performed a multivariate analysis and identified as poor prognosticators for long-term survival after DCRT the following; low baseline performance status, advanced tumor stage, and radiation dose <60 Gy [74]. Takeuchi reported a significantly worse median overall survival for EP (14.7 versus 35.1 months, *p* = 0.01), although in this study a higher percentage of EP discontinued treatment (58% vs. 17%, *p* < 0.001), mostly for hematologic toxicity [73].

Progression-free survival was assessed in three studies [19,27,72]. Vöncken et al. was the only one to report a significant difference, with a median of 20.5 months for EP versus 7.4 months for YP (*p* = 0.01); in this study, although initial tumor stage was comparable between EP and YP, the elderly had a better survival and a significantly longer progression-free interval after DCRT (62.3 vs. 11.7 months) [27].

In summary, DCRT is the most frequently offered treatment to EP with esophageal cancer. There is some evidence to support higher rates of severe hematologic and pulmonary toxicity for EP, however clinical response to treatment is at least as good if not better than YP. Concurrent DCRT is associated with the maximum survival benefit, but also higher acute toxicity compared to sequential DCRT and radiation only.

**Table 4:** Definitive Radiochemotherapy (DRCT) for elderly patients (EP) vs. younger patients (YP) with oesophageal cancer.

Author, Year [Reference]	EP Definition (Years)	No of EP (%)*% of SCC*	DRCT Details	cCR%	Hematologic Toxicity, % (>Grade II)	Pulmonary Toxicity, % (>Grade II)	Esophagitis% (>Grade II)	Progression-Free Survival	Survival Overall
				EP/YP	EP/YP	EP/YP	EP/YP	EP/YP	EP/YP
Jingu, 2020 [77]	80	358 (15.3) *96.1*	-	-	-	-	-	-	13/52 * 5-year, (%)
Vöncken, 2017 [27]	70	76 (30) *33*	Carbo-Taxol + 50 Gy	48/31	-	-	-	20.5/7.4 *	23.6/13.1 *
Münch, 2017 [19]	75	32 (45) *81*	5FU/Cisplatin + 7–60 Gy	-	13/29	-	-	10/9 median, (mo)	16/20 median, (mo)
Xu, 2017 [72]	80	56 (20) *29*	5FU/Taxane + 45–50.4 Gy	78/56 *	4/12	11/0 *	16/14	58/56 5-year (%)	28/23 median, (mo)
Davies, 2010 [14]	70	106 (45) *51*	5FU/Cisplatin + 50 Gy		22/19	-	7/7	-	22/21 median, (mo)
Takagawa, 2008 [25]	75	19 (9) *84*	5FU + 60 Gy	66/67	-	-	-	-	15/10 median, (mo)
Takeuchi, 2007 [73]	70	33 (19) *100*	5FU/Cisplatin + 60 Gy	64/63	70/50 *	-	3/9	-	15/35 * median, (mo)
Tanisada, 2000 [74]	75	123 (22) *NA*	-	-	-	-	-	-	9/11 5-year, (%)

DCRT = Definitive Chemoradiation, EP = Elderly patients, YP = Younger Patients, SCC = Squamous Cell Carcinoma, Ccr = clinical Complete Response, NA = Not Available. Significant *p*-values < 0.05 are indicated with an asterisk *. *Italics* indicate the percentage of SCC tumors in all included studies.

### 3.6. Endoscopic Treatment of Early Stage Cancer

Only two comparative studies between EP and YP were found on endoscopic submucosal dissection [78,79] for early esophageal cancer and found no significant differences in procedure-related complications. Ishii et al. report a 0% rate of complications in both groups [79], whereas Kikuchi et al. [78] found respectively 8% and 27% overall complications; emphysema was reported in 0% vs. 24% for EP vs. YP, bleeding in 0% vs. 4%, and mediastinitis in 8% vs. 4% with none of these differences being significant. R0 resection was achieved in 77–84% of EP and 59–86% of YP, with no significant differences in both studies. Median length of hospital stay was also comparable, 5–8 days for EP and 6–9 days for YP. According to the limited available data, endoscopic submucosal dissection for early-stage tumours thus seems to yield similar results in EP as in YP.

### 3.7. Quality of Life

Two studies reported a significantly higher rate of EP discharge to a nursing facility rather than the patients’ home after esophagectomy: in the study of Stahl [56] who defined EP as >80 years, 43.9% of EP versus only 6% of YP were discharged to a nursing facility after surgery (*p* < 0.0001), whereas Finlayson et al. [54] reported 54% of EP versus 84% of YP being discharged at home postoperatively (*p* < 0.001). Cavallin et al. [66] reported long-term QoL after esophageal cancer surgery according to age. Using the validated EORTC tool, they found that global QoL was similar for EP (>70 years) and YP over time, with EP having equivalent functional outcomes (dyspnea, dysphagia, fatigue) to YP and this, in spite of a worse baseline functional status in EP. Johansson et al. in an older study, reported significantly higher rates of dysphagia at 6 months for EP versus YP (26% vs. 6%, *p* = 0.032), although absolute weight loss was similar (5kg) [80].

Ten studies reported a longer postoperative hospital stay for EP compared to YP, from 1 to 6 days [20,38,41,43,45,46,47,48,56,66]. Two authors assessed the financial burden of hospital stay for EP and YP, and they both found a significantly higher cost for EP [48,56]. Two studies report a clear volume effect in esophagectomy for EP (with a less pronounced effect in YP), with lower morbidity and mortality rates for EP treated in a high volume center [42,52].

In summary, EP are at higher risk of loss of autonomy and inability to return home after esophagectomy, although their long-term QoL does not seem to be worse than YP. Treatment in a high volume center is associated with lower postoperative morbidity and mortality for EP.

## 4. Discussion

With the ongoing aging of the population, health-care practitioners will be more and more confronted with elderly patients (EP) with esophageal cancer. The current review confirmed that one out of three patients (30%) with esophageal cancer was >70 years, and one out of five (18%) over 75 years old. Although immediate postoperative morbidity and mortality seems increased for EP compared to YP, surgical results are far from prohibitive. Multimodal treatment and surgery offer the best chances of curative treatment in the elderly population, whereas definitive chemoradiation is also associated to a significant survival benefit compared to best supportive care.

EP represent a heterogeneous population, with a variable degree of baseline comorbidity and performance status, reduced physiological reserves and capacity to endure stress compared to YP [60,81]. Lagarde et al. in 2008 [82] as well as a recent analysis of the American Society of Thoracic Surgeons Database identified age as an independent predictor of major morbidity after esophagectomy [83]. One might concede that age independently increases the expected complication rate, but at the same time with older age often comes a decaying cardiac, pulmonary and peripheral vascular status. Several authors have reported both an impaired baseline cardiac/pulmonary condition and increased cardio-pulmonary complications for EP after esophagectomy [31,33,34,37,38,40,41,43,44,45,46,48,50,52,65].

Esophageal cancer surgery is still associated with high rates of postoperative morbidity, reaching 60% even in expert centers [84]. This morbidity seems to translate to higher mortality rates for EP compared to YP in several studies [10,25,29,32,34,36,37,39,40,43,44,47,48,54,55,56,57,58]. Markar et al. in a 2013 meta-analysis reported a 2 fold risk for postoperative death for patients >70 years [6], while Schlottmann et al. later reiterated this finding, with a higher mortality for patients >70 years and a linear increase of the death rate with increasing age (2.5% in 50 years versus 5.4% in 70 years, and 7.0% in 80 years) [48]. But can these mortality rates be seen as prohibitive? Hardly so, if we consider recently published benchmarks for mortality after esophagectomy (performed in optimal conditions, healthy patients and expert centers), estimated between 2.4% (30-days) and 4.5% (90-day) [84]. A very interesting concept in this regard seems to be the ‘failure to rescue’, meaning failure to avoid death in a patient presenting complications. Liou et al. identified age >75 years along with Afro-american ethnicity, ASA class 4–5 and major complications as independent predictors of failure to rescue after esophagectomy [85]. Schweigert found mortality 8.5 times higher in EP compared to YP presenting an anastomotic leakage [37], whereas according to a previous study failure to recognize and proactively treat an infectious or pulmonary complication in EP was an independent predictor of failure to rescue [86].

Definitive chemoradiation (DRCT) is the most frequently used treatment modality in EP with esophageal cancer [87], yielding excellent outcomes especially for the squamous cell subtype (SSC) [88]. Although rates of SSC in EP varied from 29 to 100% in the included studies, no differences in histological type were observed in comparison to YP. In particular, in the study by Xu et al. [72], SCC was the histological type in 29% EP and 24% YP (*p* = 0.707). Thus, the significant difference in clinically complete response in favor of EP cannot be attributed to more favorable squamous cell histology. Similarly, Vöncken et al. [27] report a longer progression-free survival for EP, although SCC rates were similar (33% EP vs. 36% YP, *p* = 0.88), with comparable rates of complete clinical response. Although a robust explanation of this ‘paradox’ of better outcomes in EP cannot be safely provided by available data, some assumptions can be made. Slower baseline cell metabolism and proliferation in the aging population could provide a more sustained response to cytotoxic treatment. As such, further research on the subject is warranted to elucidate this complex pathophysiological background.

Severe toxicity was reported in similar rates in EP as in YP [25,27,72,73], while regimens at a reduced dose have also been studied in EP with very promising results as far as efficacy and treatment-related side effects are concerned [76]. Similarly, neoadjuvant chemo(radio)therapy is often underused in EP by fear of increased toxicity and postoperative morbidity. Lorenzen et al. assessed patients > 65 years and found that by omitting docetaxel from the FLOT regimen, toxicity and postoperative morbidity were significantly reduced, with only a trend towards shorter progression-free survival [89]. Given the remarkable efficacy of this regimen in the treatment of gastroesophageal adenocarcinoma, clinicians should be familiar with this option for EP. Unfortunately, the CROSS study for neoadjuvant chemoradiation defined age > 75 among the exclusion criteria [90] whereas the earlier OEO2 [91] and MAGIC [92] trials had included very few EP with esophageal cancer to allow specific conclusions. In a recent large-scale study of the CROSS regimen in patients >75 years, increased overall survival was observed after neoadjuvant treatment and surgery compared to surgery alone or DRCT, with no differences in postoperative mortality [87]. Although the optimal neoadjuvant treatment modality has yet to be defined, the Swedish NeoRes trial suggested increased severity of postoperative complications after neoadjuvant chemoradiotherapy compared to chemotherapy [93]. Thus, avoiding radiotherapy might be an interesting option for elderly and frail patients to optimize postoperative outcomes. Of note, one study in the present review [27] reported better histologic response after NATS in EP. One could hypothesize that baseline tumor biology might ‘slow down’ or become less aggressive as age advances, however as data on the subject are extremely scarce, caution is needed before establishing potential conclusions on this complex issue.

In most of the above studies, overall survival is the key outcome assessed, although it seems a rather insufficient endpoint for patients having already exceeded median life expectancy. However, EP after curative treatment could still significantly improve their survival, from 3 to 24 months compared to palliative chemo- or radiotherapy or best supportive care [12,16]. As we are often reminded when treating EP, what matters even more than these additional months to live is the quality of life (QoL) we might expect. Though it needs to be pointed out that progression of untreated esophageal cancer can itself lead to a significant decay in patients’ QoL, esophagectomy induces a significant and durable decrease on patients’ quality of life, from the immediate postoperative period up until 24–48 months later [94]. No data exist to support worse QoL or severity of symptoms after oesophagectomy for EP [8,66,94]. Cavallin et al. in the only study specifically assessing EP, found similar QoL between EP and YP both upon discharge and at 3 months postoperatively [66]. However, as age increases the risk of a nursing care facility placement after the operation rises accordingly [54,56].

The future of esophageal cancer management in EP patients should thus, be marked by the need not to systematically exclude this population from potentially curative strategies, but to tailor treatment according to the individual characteristics, treatment-related risks, as well as expected oncologic outcomes. Except for the potential modifications to the oncologic treatment mentioned above [89], type and extent of surgery need special consideration. Although many of the retrieved studies include open thoracotomy, minimally invasive surgery is increasingly gaining acceptance as the standard of care compared to the open approach, offering better postoperative outcomes and specifically reduced pulmonary morbidity [95], and a lesser impact in long-term quality of life compared to the open approach [96]. Sugita et al. recently studied the feasibility of minimally invasive approach specifically in EP, demonstrating comparable postoperative morbidity and mortality to younger patients [97]. Such technical advances may be expected to provide lower postoperative morbidity and a subsequent better QoL for EP, which represent currently the major limiting factor for their access to curative treatment. Furthemore, avoiding transthoracic approach altogether, if the tumor location allows it, could be a valuable treatment alternative for frail EP. Extended transhiatal gastrectomy demonstrated similar long-term survival as was recently accessed in a French series of Siewert II tumors, demonstrating comparable long-term survival to transthoracic esophagectomy, although negative resection margins might be more difficult to obtain [98]. As such, optimizing surgical strategy to the least invasive approach might be of particular interest in EP, as their reduced physiological reserve amplify the impact of surgical stress and postoperative morbidity.

Adequate initial assessment of EP with esophageal cancer is mandatory to optimize treatment options and ensure patients’ will and capacity to undergo treatment. For instance, the G8 score can help identify frail patients needing the intervention of a geriatric oncologist [99], as specifically suggested in a comprehensive review of gastric cancer surgery in elderly patients [100]. Montroni et al. recently published the wide panel of geriatric assessment tools used for the elderly oncologic patient, underlining the importance of preoperative physical but also nutritional, cognitive and functional screening in EP before major cancer surgery [101]. This all adds to the increasing body of evidence that patient’s frailty rather than their biological age should direct patient management, especially when both the disease and its treatment may have a severe and persistent impact on all aspects of QoL.

There are some limitations in our study that need to be mentioned. Direct comparison of results in a quantitative synthesis was not performed, mostly because of the heterogeneity in defining and recording complications, even for the hard outcomes such as survival and postoperative mortality. Despite this, we tried to give a depiction of all retrieved data as accurately and concisely as possible, by means of a pooled analysis of results for each endpoint. The absence of specific consideration of histological type in most included studies rendered unfeasible separate subgroup analyses for adenocarcinoma and SCC. However, this cannot be considered a major bias in the interpretation of our results, for the following reasons: although a different biologic basis may exist for each histological type, current treatment options are often identical. This includes type of surgery, neoadjuvant treatment, as well as definitive chemoradiotherapy (DCRT). The principal difference lies in the wider acceptance of DCRT in SCC, with a purported higher probability of complete response, although ongoing studies assess DCRT and a ‘watch-and-wait’ attitude even for adenocarcinoma. This is in accordance with the results of our systematic review, illustrating that even in studies on DCRT, both types are included. Furthermore, several of the reported outcomes for elderly patients in our study, not taken into consideration in any previous reviews on the subject (i.e., access to surgery, postoperative complications, mortality, chemoradiation toxicity and quality of life), are completely independent from histological type. In addition, baseline comorbidity status was not always clearly defined for elderly and younger patients and cannot be presumed to be similar. Although this might be considered a source of confounding, we strongly believe it illustrates real-life practice and should be taken into account during the decision-making process for EP. Finally, all available studies being retrospective, the Newcastle-Ottawa quality tool was used to select the highest quality of available evidence on the subject.

In conclusion, advanced age in itself should not be prohibitive for curative esophageal cancer treatment. A well-informed discussion of all available therapeutic options should be undertaken, after a thorough evaluation of the physical and social function of the geriatric patient, and sincere consideration of patients’ wishes and expectations of the offered treatment.

## 5. Conclusions

Although the peak incidence of esophageal cancer is seen in patients >70 years, elderly patients (EP, >70 y) are often undertreated even for curable stages of the disease. This systematic review suggests that any treatment modality may provide a survival benefit for EP compared to best supportive care.

Definitive chemoradiation is the most frequent treatment modality offered to EP. Although higher rates of hematologic and pulmonary toxicity may be observed, clinical response to treatment is similar to younger patients, offering EP a valid option for disease control and improved survival. Neoadjuvant treatment followed by surgery is feasible in EP, although increased postoperative cardiopulmonary morbidity may be expected, needing cautious choice of the chemoradiation regimen. Surgical complications do not differ significantly with younger patients; however, ‘failure to rescue’ and postoperative mortality appear more frequent in EP, without reaching prohibitive rates. Importantly, EP are at higher risk of autonomy loss after esophageal cancer surgery, although their long-term QoL does not seem to be worse than YP. Treatment in a high volume center is associated with lower postoperative morbidity and mortality for EP.

## Data Availability

Data supporting reported results are available from the corresponding author upon request.

## Appendix A

Search strategy in Pubmed

((((((“esophageal neoplasms”[MeSH Major Topic]) or “esophageal cancer”[Title/Abstract]) or esophageal cancer[Title/Abstract])) or esophagectomy)) and (((((elderly[Title/Abstract]) or older[Title/Abstract]) or age)) or geriatric)
